# Development, validation, and clinical assessment of a liquid chromatography-tandem mass spectrometry serum assay for per- and polyfluoroalkyl substances (PFAS) recommended by the National Academies of Science, Engineering, and Medicine (NASEM)

**DOI:** 10.1007/s00216-024-05519-y

**Published:** 2024-09-13

**Authors:** Wen Dui, Michael P. Smith, Sarah H. Bartock

**Affiliations:** Quest Diagnostics, 14225 Newbrook Drive, Chantilly, VA 20151 USA

**Keywords:** Per- and polyfluoroalkyl substances, PFAS, PFOA, PFOS, NASEM, LC–MS/MS

## Abstract

Per- and polyfluoroalkyl substances (PFAS) are widely used in industry, residential, and consumer products. Studies have shown associations between high PFAS exposure and adverse health effects. In 2022, the National Academies of Science, Engineering, and Medicine (NASEM) published *Guidance on PFAS Exposure, Testing, and Clinical Follow-up* providing laboratory and clinical direction. The Guidance suggests nine PFAS should be measured in serum or plasma specimens and summed to provide a total PFAS concentration using a NASEM-recommended method. Follow-up clinical recommendations are based on the calculated PFAS NASEM summation. We developed and validated a liquid chromatography-tandem mass spectrometry (LC–MS/MS) method in accordance with NASEM recommendations but distinguished by the ability to separate closely related structural isomers. As part of our validation, PFAS prevalence was evaluated in a population survey comprised of clinical donor and remnant specimens (*n* = 1023 in total). In this study, 82.2% of the specimens had PFAS NASEM summations of 2 to < 20 ng/mL and 2.5% had a summation ≥ 20 ng/mL. The median PFAS NASEM summation was 4.65 ng/mL in this study, lower than the 7.74 ng/mL median observed in the 2017–2020 Centers for Disease Control and Prevention, National Health and Nutrition Examination Survey (*n* = 3072). This lower median PFAS NASEM summation may reflect a decline in PFAS population levels over time or sample population exposure differences.

**Graphical Abstract:**

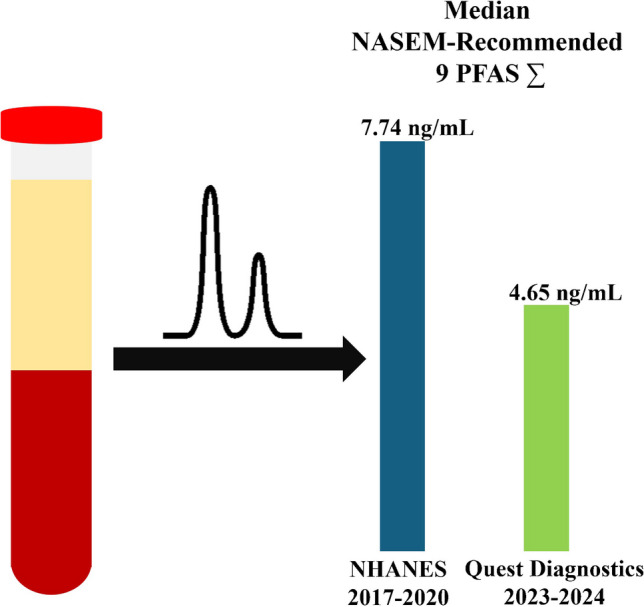

**Supplementary Information:**

The online version contains supplementary material available at 10.1007/s00216-024-05519-y.

## Introduction

Per- and polyfluoroalkyl substances (PFAS) are a class of manufactured chemicals that have been widely used in industrial, residential, and consumer products for more than 80 years [1]. With their unique chemical properties, oil and water repellency, friction reduction, and temperature resistance, PFAS have made their way into countless products, bringing benefits to everyday life with comforts and conveniences that have silently shaped daily life for decades [2].

Unfortunately, the same characteristics that make PFAS useful are now causing concerns. All PFAS share one common trait: a strong carbon–fluorine bond that is extremely resistant to typical environmental degradation processes [3–8]. Consequently, PFAS linger in the environment and have been termed “forever chemicals,” which globally contaminate water, air, and soil [9, 10]. PFAS have been found in the water and soil of all 50 states of the USA [11, 12]. As a result of the extensive production, the persistence of PFAS in the environment leads to accumulation in human bodies when exposure exceeds elimination rate. The Centers for Disease Control and Prevention (CDC), National Health and Nutrition Examination Survey (NHANES) have been monitoring PFAS prevalence since 2013, and 2017–2020 study data suggest that nearly all people in the USA may have at least one PFAS in their body. With the cessation of production of the most common PFAS, population levels appear to be declining [13–16].

Elevated PFAS exposure is associated with immune suppression, decreased antibody response, dyslipidemia, elevated cholesterol, decrease infant and fetal growth, pre-eclampsia, high blood pressure during pregnancy, increased risk of kidney cancer, thyroid disease, changes in liver enzymes, and other human metabolic pathways [17–39]. In response, several federal agencies have worked to summarize evidence and provide recommendations for clinicians, including the CDC’s Agency for Toxic Substances and Disease Registry (ATSDR) in 2019 [40]. In 2022, NASEM published *Guidance on PFAS Exposure, Testing, and Clinical Follow-up*, which lists PFAS species, reference ranges, and exposure reduction recommendations; evaluates and categorizes evidence for adverse health effects; and provides clinical guidance for follow-up with patients after PFAS testing [11]. In January 2024, CDC published *PFAS Information for Clinicians-2024* and urged more blood testing for PFAS [41].

To address the critical need for reliable PFAS measurement in clinical laboratories, our objective was to use the NASEM and CDC documents as a guide to develop and validate a liquid chromatography-tandem mass spectrometry (LC–MS/MS) method to detect and quantify nine PFAS. Other methods with more PFAS species included have been published in environmental matrices and human specimens [3, 16, 19, 28]. However, these previous methods have longer run times leading to long turnaround times in clinical settings, low detection frequency for most of the PFAS species especially in human serum beyond the nine recommended by NASEM, and extensive cleanup sample extraction procedures. As these aspects bring challenges for clinical PFAS testing, we aimed to develop a simpler method for the nine PFAS, including closely related structural isomers, in human serum and plasma specimens. Once validated, we sought to test the clinical applicability of the method by examining the prevalence of the various PFAS and total PFAS levels, calculated per NASEM-recommended summation, in > 1000 specimens from donors and patients. We also sought to verify that PFAS levels may be declining on a population basis.

## Materials and methods

### Human serum and plasma matrices

Mass Spect Gold Human Serum, Ultra-Low Hormones & Steroids was purchased from Golden West Diagnostics (Temecula, CA) and was confirmed negative for all analytes at the assay’s limits of quantification (LOQs) prior to calibrator (CAL) and quality control (QC) preparation.

### Reference standards and reagents

All standard materials and deuterated internal standards (Table S1) as well as other PFAS and PFAS-related compounds were purchased from Wellington Laboratories (Ontario, Canada). LC–MS-grade methanol (J.T. Baker) and LC–MS-grade acetonitrile were from Millipore Sigma (Saint Louis, MO). Reagent-grade formic acid (≥ 95%) (Honeywell Fluka) and ammonium acetate crystalline were from Fisher Scientific (Fair Lawn, NJ). Water was purified with a HYDRO Ultrapure Water System (EVOQUA, SILICA 0 ppm).

### Preparation of standard solutions

Individual PFAS standard solutions were prepared in blank serum at concentrations of 0.05, 0.1, 0.2, 0.5, 1.25, 2.5, 5, 12.5, and 25.0 ng/mL (0.024, 0.049, 0.10, 0.24, 0.61, 1.22, 2.44, 6.11, and 12.22 ng/mL for branched perfluoro-5-methylheptane sulfonate (br PFOS-P5)). QC materials at LOQs and low, medium, and high levels were prepared across the linear dynamic range for each analyte in blank serum. A working internal standard solution was prepared in 75:25 methanol:water (volume/volume). All stock concentrations were corrected if any salt form provided from Wellington Laboratories before preparing solutions.

### Instrumentation

PFAS data were collected on a Sciex Triple Quad 7500 system mass spectrometer equipped with D jet ion guide, OptiFlow Pro ion source, and E Lens probe (Sciex, Framingham, MA), interfaced to a Shimadzu 40DXR system with 2 LC-40DXR pumps, a CTO-40C column oven, and a SIL-40XCR autosampler (Shimadzu Corporation, Kyoto, Japan). Sciex OS was utilized for data acquisition and processing. PFAS can be present in traditional PTFE-coated LC materials; therefore, instrumentation for this method also included the Sciex LC PFAS kit which contained PFAS-free solvent inlet filters, transfer lines, and tubing throughout the system.

### Clinical specimens

 In addition to 106 donor specimens, 917 deidentified clinical remnant specimens from different Quest Diagnostics laboratories across west coast, northeastern, and mid-Atlantic regions were included as a PFAS surveillance study (*n* = 1023 specimens in total).

### Procedures

#### Specimen collection

Paired red-top serum and lavender-top plasma (*n* = 97) and paired serum from serum separator tubes (SST) and red-top tubes (*n* = 12) were collected from the same individuals under IRB approval. To avoid potential assay interferences, collection devices containing polytetrafluoroethylene (Teflon® (Chemours, Wilmington, DE)) and polyvinylidene fluoride were avoided. To confirm standard phlebotomist serum specimen collection materials were PFAS-free, water was used as a test matrix and collected through butterfly devices, into serum vacutainer tubes, allowed to sit for the standard clotting time at room temperature, centrifuged, transferred into polypropylene tubes, sent through typical overnight laboratory transportation processes, and extracted the next day as serum specimens.

#### Specimen preparation

Serum or plasma specimens (100 µL) were diluted with 50 µL of isotopically labeled internal standards followed by 300 µL of 1% formic acid in acetonitrile to precipitate proteins. The specimens were centrifuged for 10 min at 2500 relative centrifugal force (RCF) and 250 µL of the supernatant was transferred to a plate for LC–MS/MS injection.

#### LC–MS/MS

A delay column (3 μm, 50 × 3 mm) (Phenomenex, Torrance, CA) was installed before the autosampler, to counteract endogenous environmental PFAS interferences present in the system’s mobile phases. A binary gradient of mobile phase A (10 mM ammonium acetate in water) and mobile phase B (10 mM ammonium acetate in 75% acetonitrile, 25% methanol) was employed with a total run time of 14 min at 0.5 mL/min. Chromatographic resolution was achieved with a gradient that ramped from 40 to 48% mobile phase B over a minute, then to 50% mobile phase B over 5 min on a porous C18 column (3 µm, 100 × 2 mm) (Phenomenex, Torrance, CA). Autosampler and column oven temperatures were 15 °C and 30 °C, respectively. Data were collected on a Sciex Triple Quad 7500 system using electrospray ionization (ESI) in negative mode. Compound-specific MS/MS parameters and source settings were optimized via direct infusion of 5 to 20 ng/mL solutions in methanol at 7 µL/min (Table 1). Optimized source parameter were as follows: 45 psi gas 1, 70 psi gas 2, 45 psi curtain gas, 425 °C source temperature, 14 CAD gas, − 1800 V ion spray voltage. Scheduled multiple reaction monitoring (sMRM) scan mode was employed and the detection time window was 60 s. Two sMRMs were collected for each analyte and an ion ratio was determined. One sMRM was collected for each internal standard.

**Table 1:** Mass spectrometer parameters for PFAS in serum (quantifier on the top and qualifier below)

Analyte	Q1 mass (m/z)	Q3 mass (m/z)	CE (V)	CXP (V)	Q0D (V)
Linear PFOS	499	80	− 106	− 10	− 100
	499	99	− 86	− 10	− 140
br PFOS-P5	499	80	− 118	− 9	− 100
	499	230	− 52	− 15	− 100
br PFOS-P6	499	80	− 107	− 13	− 100
	499	230	− 56	− 12	− 100
MPFOS^a^	503	80	− 106	− 10	10
Linear PFOA	413	369	− 25	− 15	10
	413	169	− 35	− 15	10
br PFOA-P5	413	219	− 12	− 15	10
	413	119	− 28	− 15	10
MPFOA^a^	417	372	− 15	− 15	10
PFHxS	399	80	− 50	− 10	10
	399	119	− 43	− 10	10
MPFHxS^a^	403	103	− 78	− 10	10
PFNA	463	219	− 24	− 10	10
	463	169	− 26	− 10	10
MPFNA^a^	403	103	− 15	− 15	10
PFDA	513	219	− 26	− 15	10
	513	269	− 26	− 15	10
MPFDA^a^	515	470	− 17	− 15	10
PFUnDA	563	519	− 32	− 15	10
	563	269	− 18	− 15	10
MPFUnDA^a^	565	520	− 17	− 15	10
MeFOSAA	570	419	− 29	− 15	10
	570	483	− 23	− 15	10
d3-N-MeFOSAA^a^	573	419	− 29	− 15	10

^a^Internal standards listed after corresponding analyte(s)

#### Quantitation

Following NASEM recommendations, a summed concentration of nine PFAS compounds (MeFOSAA, PFHxS, linear PFOA, branched PFOA, PFDA, PFUnDA, linear PFOS, branched PFOS, and PFNA) was calculated (see Table S1 for a full list of compound abbreviations), including utilizing a 0.07 ng/mL value for PFAS analytes with levels below the assay-defined 0.1 ng/mL cutoff for all analytes [11]. Br PFOS-P5 and br PFOS-P6 concentrations were summed and treated as a single analyte, branched PFOS.

#### Validation

Linearity, accuracy, specificity, imprecision, carryover, matrix effects, dilution integrity, and analyte stability were evaluated according to Clinical Laboratory Improvement Amendment 1988 regulations for laboratory-developed tests (LDTs). Guidelines from the Clinical and Laboratory Standards Institute (CLSI) and the Approved American National Standard/Academy Standard Board (ANSI/ASB) were also followed [42–44].

Linearity assessment for each analyte utilized nine concentrations on five separate days. LOQs were defined as the lowest analytical measurement range (AMR) concentration. Imprecisions were determined over 5 days from five replicates at four QC concentrations: LOQ, 125% cutoff, 40% upper limit of linearity (ULOL), and 80% ULOL. Imprecision for each level (*n* = 25) was expressed as mean, bias (%), within-run CV (%), and between-run CV (%).

Because no available patient specimens were found to be devoid of all analytes, matrix effects were evaluated by comparing calculated concentrations of water-diluted samples to undiluted samples (target), expressed as a percent difference. Therefore, for the matrix effect study, low PFAS-positive patient specimens were screened and saved. The matrix effect at a low-positive level was evaluated by spiking two patient specimens with PFAS at 0.125 ng/mL (0.063 ng/mL for branched PFOS-P5) and then diluting them twofold with water. The matrix effect at a high-positive level was evaluated by spiking two patient specimens with PFAS at 20.0 ng/mL (9.8 ng/mL for branched PFOS-P5) and then diluting them two-, five-, and tenfold with water. All the diluted results were compared to original (undiluted) results and the percent difference was calculated.

Carryover was assessed by injecting negative QC (extracted blank serum with internal standards) immediately after a sample containing analytes at the ULOL level. Absence of carryover was documented by failure of LOQ criteria.

Dilution integrity of two-, five-, and tenfold was assessed with 40% ULOL, 80% ULOL, and five times 80% ULOL diluted by blank serum (Golden West Diagnostics).

Specificity was evaluated by challenging the method with related and non-related compounds. Non-related interferences, including prescription and over-the-counter medications, and drugs of abuse (Table S2), were evaluated by fortifying 1000 ng/mL into negative serum matrix and LOQ samples. Available PFAS and PFAS-related compounds (Table S3) were tested individually by fortifying 10 ng/mL of drugs into negative serum matrix and LOQ samples. Absence of interference in the LOQ samples was demonstrated by achieving analyte relative retention time within 2% of the mean calibrator retention time and transition peak area ratios within ± 20% (linear PFOA, PFNA, MeFOSAA, PFDA, and PFUnDA) or 30% (PFHxS, linear PFOS, branched PFOS, and branched PFOA) of the mean calibrator peak area ratios following the Clinical and Laboratory Standards Institute (CLSI) guidance.

Analyte storage stability was evaluated with ten serum and plasma donor specimens fortified with 1 ng/mL, 5 ng/mL, and 10 ng/mL (0.49 ng/mL, 2.44 ng/mL, and 9.77 ng/mL for br PFOS-P5) stored at room temperature, refrigerated, and frozen conditions. Specimens were initially extracted (considered day 0), then aliquoted into the test groups and then extracted at different time points, and the concentrations were compared with day 0. The stability of three freeze/thaw cycles was also evaluated. Autosampler stability was assessed by reinjecting specimens after 72 h and comparing the calculated concentration to the initial concentration.

## Results

### Validation

The described method met all study design criteria. The AMR was 0.05 to 25.0 ng/mL for PFHxS, linear PFOS, br PFOS-P6, linear PFOA, br PFOA-P5, PFNA, PFUnDA, PFDA, and MeFOSAA and 0.02 to 12.2 ng/mL for br PFOS-P5. The reporting cutoff for all analytes was administratively set to 0.1 ng/mL. A quadratic regression with 1/*x*^2^ weighing was used for the calibration curves of all analytes, with the exception that 1/*x* was used for PFUnDA. The seven linear isomer analytes utilized matched internal standards for quantitation, while the three branched isomers used the corresponding linear analog as internal standards. All calibration curve correlation coefficients (*R*^2^) were > 0.998. Accuracy bias across the AMR was evaluated over 5 days using nine concentrations, including LOQ and ULOL levels, with bias ranging from − 12 to 5% (Table 2). Imprecision was evaluated with five replicates of four QC concentrations across the linear range over 5 days. Within-run CV was from 0.76 to 6.10% (*n* = 5) and between-run CV was from 0.92 to 9.99% (*n* = 25) (Table 3).

**Table 2:** Accuracy at the limits of quantification (LOQ) and upper limit of linearity (ULoL) and linearity (*R*^2^) for PFAS in serum (*n* = 5)

Analyte	LOQ/ULoL	Target Conc. (ng/mL)	Mean (ng/mL)	%CV	% Deviation	*R*^2^
PFHxS	LOQ	0.05	0.048	4.00%	97%	0.9999
	ULoL	25	25.240	3.05%	101%	
Linear PFOS	LOQ	0.05	0.048	1.06%	97%	0.9999
	ULoL	25	25.730	3.80%	103%	
br PFOS-P5	LOQ	0.024	0.024	3.52%	98%	0.9999
	ULoL	12.22	12.280	4.08%	101%	
br PFOS-P6	LOQ	0.05	0.049	4.77%	98%	0.9999
	ULoL	25	26.094	7.42%	104%	
Linear PFOA	LOQ	0.05	0.048	2.18%	95%	0.9999
	ULoL	25	24.549	5.59%	98%	
br PFOA-P5	LOQ	0.05	0.047	3.67%	95%	0.9999
	ULoL	25	24.894	5.64%	100%	
PFNA	LOQ	0.05	0.050	1.35%	100%	0.9999
	ULoL	25	25.591	5.27%	102%	
MeFOSAA	LOQ	0.05	0.049	1.39%	98%	0.9999
	ULoL	25	25.754	3.99%	103%	
PFDA	LOQ	0.05	0.049	4.44%	98%	0.9999
	ULoL	25	25.022	5.85%	100%	
PFUnDA	LOQ	0.05	0.044	7.98%	88%	0.9986
	ULoL	25	26.214	7.98%	105%

**Table 3:** Mean bias and imprecision results for PFAS in serum at 4 quality control concentrations

Analyte	Target Conc. (ng/mL)	Mean (ng/mL) (*n* = 25)	Bias (%)	Within-run CV (%)	Between-run CV (%)
Linear PFOS	0.05	0.049	− 2%	1.93%	2.06%
	0.125	0.126	1%	1.15%	1.16%
	10	10.1	1%	1.13%	1.18%
	20	20.8	4%	1.32%	1.34%
br PFOS-P5	0.024	0.024	0%	1.87%	3.04%
	0.0625	0.062	0%	1.40%	1.72%
	4.888	4.98	2%	2.41%	2.91%
	9.776	10.2	4%	3.00%	3.30%
br PFOS-P6	0.05	0.049	− 2%	2.30%	3.93%
	0.125	0.127	2%	1.98%	2.13%
	10	10.2	2%	2.30%	2.83%
	20	21.3	6%	2.70%	3.11%
Linear PFOA	0.05	0.048	− 4%	4.53%	5.27%
	0.125	0.126	1%	3.30%	3.41%
	10	9.93	− 1%	3.54%	3.36%
	20	20.4	2%	4.39%	4.47%
br PFOA-P5	0.05	0.049	− 2%	4.17%	4.13%
	0.125	0.126	1%	3.31%	3.05%
	10	10.1	1%	3.10%	3.64%
	20	20.9	4%	2.92%	3.03%
PFHxS	0.05	0.048	− 4%	3.42%	3.26%
	0.125	0.125	0%	2.30%	2.19%
	10	10.1	1%	1.47%	1.68%
	20	20.7	3%	1.54%	2.25%
PFNA	0.05	0.049	− 2%	2.41%	2.25%
	0.125	0.127	2%	1.80%	1.71%
	10	10.1	1%	1.78%	2.13%
	20	20.8	4%	1.71%	2.74%
PFDA	0.05	0.049	− 2%	2.90%	2.88%
	0.125	0.128	2%	3.12%	3.08%
	10	9.99	0%	1.68%	1.78%
	20	21.1	5%	2.35%	3.34%
PFUnDA	0.05	0.044	− 12%	4.81%	9.99%
	0.125	0.129	3%	4.28%	5.69%
	10	9.62	− 4%	4.70%	6.37%
	20	19.63	− 2%	6.10%	8.87%
MeFOSAA	0.05	0.049	− 2%	1.80%	1.83%
	0.125	0.127	2%	0.89%	0.92%
	10	10.0	0%	0.76%	0.92%
	20	20.93	5%	1.27%	1.49%

No significant matrix effects were observed in this assay; all differences were within 20%, with an average matrix effect of 0.39% at the low PFAS level and − 6.54% at the high PFAS level (Table S4). No assay carryover was observed in a negative QC following the ULOL injection. Dilution integrity was verified at two-, five-, and tenfold with 40% ULOL and 80% ULOL, and five- and tenfold with the five times 80% ULOL diluted by blank serum; all dilutions achieved accuracy within 20%.

Specificity was evaluated with 146 common prescription and nonprescription drugs at 1000 ng/mL in both negative serum matrix and LOQ samples; no interferences were observed and none of the challenges caused transition ratio or quantification failure in LOQ samples. Additionally, 54 PFAS and PFAS-related compounds (Table S1 and Table S3) were challenged. No interferences were observed for PFHxS, linear PFOA, PFNA, PFUnDA, PFDA, and MeFOSAA. Some interferences from PFAS isomers were observed during early development for br PFOS-P5, br PFOS-P6, linear PFOS, and br PFOA-P5 and mitigated to an extent with additional experimental details described below (see “PFAS isomeric resolution”).

The PFAS compounds in this method were stable, defined by average concentration changes ≤ 10%, under all conditions through the day 90 testing end point, and also showed stability with three freeze/thaw cycles and 3 days in a refrigerated autosampler (Table S5).

### PFAS isomeric resolution

#### PFOS

**Fig. 1:**
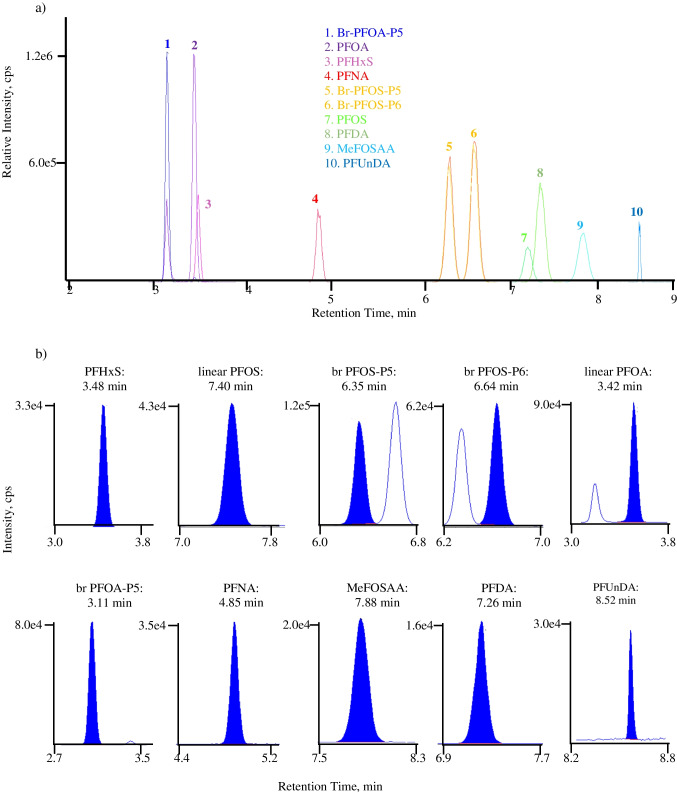
Chromatographical view of ten PFAS analytes in LC–MS/MS method, scheduled multiple reaction monitoring (sMRM) scan mode with a detection time window of 60 s (PFUnDA 40 s). (**a**) Extracted ion chromatogram (XIC) view of all analytes at 1.25 ng/mL. (**b**) Individual chromatograms of quantifier at 0.1 ng/mL, analyte name on the top followed by the retention time (min)

In this method, the two common branched PFOS isomers, br PFOS-P5 and br PFOS-P6, were quantified separately (Fig. 1) and the concentrations were summed as branched PFOS per the NASEM-recommended summation. The other available forms of monomethylated isomers (br PFOS-P3, br PFOS-P4, br PFOS-P1) and dimethylated isomers (br PFOS-P35, P45, and P55) were assessed for interference effects on linear and branched PFAS isomers.

Br PFOS-P3 coeluted with br PFOS-P5; br PFOS-P3-only spike samples showed accuracy from 75 to 87% (average 79%) with the ion ratio falling out of the 30% range established from the average calibrator ion ratio based on transitions m/z 499/80 and m/z 499/230 following the CLSI guidance. However, a sample co-spiked with br PFOS-P3 and br PFOS-P5 at 1 ng/mL demonstrated accuracy from 84 to 91% (average 88%) with the ion ratio in the 30% range. Qualifier ion ratio failure was only observed when br PFOS-P3 abundance exceeded br PFOS-P5 in the spike studies. This scenario was observed with a low frequency (< 1%) in patient and remnant sample testing with additional transition m/z 499/130 monitored for research and development purpose.

Br PFOS-P4 also coeluted with br PFOS-P5; br PFOS-P4-only spike samples showed accuracy from 116 to 136% (average 127%) with the ion ratio in the 30% range. A sample co-spike of br PFOS-P4 and br PFOS-P5 at 1 ng/mL demonstrated accuracy from 104 to 111% (average 108%) with the ion ratio in the 30% range. Further fortified challenges co-spiked with br PFOS-P3, br PFOS-P4, and br PFOS-P5 at 1 ng/mL showed accuracy from 98 to 103% (average 100%) with in-range ion ratios. The unique transition response pattern helped us finalize the quantifier as m/z 499/80 and qualifier as m/z 499/230 to report both P5 and P4 accurately in clinical specimens; when br PFOSP3 co-exists with other isomers, a failed ion ratio will occur, and it will be reported with an interference message (very rare in our clinical assessment study).

Br PFOS-P1 coeluted with linear PFOS but this inference was mitigated by adjusting MS parameters and monitoring the unique product ion (m/z 80) and shared product ion (m/z 99) [45]. When br PFOS-P1 coexisted in samples, the peak area of qualifier m/z 99 increased with no change of quantifier m/z 80 peak area; this resulted in an increased m/z 99/80 ion ratio. An initial 18 patient specimens were evaluated for this effect with > 30% (eight patient specimens) showing ion ratio failures. To reduce the br PFOS-P1 interference on linear PFOS, the Q0D setting was ramped and optimized. At Q0D − 140 V, the br PFOS-P1 response was reduced more than 95% while the linear PFOS response was maintained.

Br PFOS-P35, P45, and P55 were chromatographically separated from br PFOS-P5 and P6; therefore, no interference was observed.

#### PFOA

For branched PFOA, br PFOA-P5 is included in the calibrator and other forms of branched PFOA were evaluated as interference challenges; br PFOA-P3, P4, and P6 all coeluted with br PFOA-P5. Individual challenges of br PFOA-P3, P4, and P6 at 1.90 ng/mL, 2.2 ng/mL, and 3.1 ng/mL quantified by br PFOS-P5 using fragment ion m/z 219 showed responses of ~ 0.5%, ~ 3%, and ~ 6%, respectively, compared to target. These challenges showed ion ratios falling out of the 30% range as the qualifier fragment ion, m/z 119 responded ~ 20%, ~ 300%, and ~ 6%, respectively, compared to target. As no positive branched PFOA was observed in over 1000 authentic patient specimens tested, this interference had no effect on patient specimens in this study.

### Evaluation of specimen contamination

No PFAS contamination in the specimen collection, transportation, and storage process was observed using standard materials. Equivalency in PFAS results between different tube types, paired red-top serum, and lavender-top plasma was demonstrated by differences < 10% between the specimen types for all PFAS analytes.

### Clinical assessment of donor and patient remnant specimens

For the 1023 tested specimens, 16 (1.6%) showed all PFAS compounds less than the 0.1 ng/mL cutoffs, while 1007 (98.4%) had at least one positive individual PFAS result ≥ 0.1 ng/mL. The positivity rate (defined as ≥ 0.1 ng/mL cutoff) in descending order was > 90% for linear PFOS, linear PFOA, PFHxS, branched PFOS, and PFNA; < 50% for PFDA, PFUnDA, and MeFOSAA; and 0% for branched PFOA (Table 4).

**Table 4:** Individual PFAS prevalence study results, NHANES and Quest Diagnostics

PFAS species	NHANES, 2017–2020, *n* = 3072				Quest Diagnostics, 2023–2024, *n* = 1023			
	Concentration, ng/mL			Positive, %	Concentration, ng/mL			Positive, %
	Median	Average	Highest observed		Median	Average	Highest observed	
Linear PFOS	2.70	4.48	95.10	99.6%	1.17	1.92	43.9	98.1%
Branched PFOS	1.10	1.63	19.30	98.7%	0.70	0.95	8.20	92.5%
Linear PFOA	1.30	1.61	52.80	99.5%	0.90	1.14	1.94	97.5%
Branched PFOA	0.07	0.08	0.70	6.3%	0.07	0.07	N/A	0%
PFHxS	1.00	1.57	48.80	98.6%	0.73	1.07	27.30	95.0%
PFNA	0.40	0.65	7.00	94.8%	0.30	0.41	3.60	91.7%
PFDA	0.20	0.28	7.30	80.7%	0.07	0.16	2.90	45.4%
PFUnDA	0.10	0.18	4.80	58.5%	0.07	0.13	2.00	32.3%
MeFOSAA	0.07	0.18	4.50	49.7%	0.07	0.11	2.90	11.7%

Compared with the NHANES 2017–2020 results [13–15], our 2023–2024 individual PFAS results (Table 4) and PFAS NASEM summations (Table 5) were of the same order of magnitude. Linear PFOS, linear PFOA, and PFHxS were the most prevalent PFAS in our study as they were in the NHANES study; the median NASEM-recommended PFAS summation in our study population was 4.65 ng/mL compared to 7.74 ng/mL in the NHANES study, possibly reflecting a decline in population PFAS concentrations over time or sample population exposure differences. Linear PFOS showed the largest change, from a median 2.70 ng/mL in NHANES to 1.17 ng/mL in our data [46].

**Table 5:** PFAS NASEM summation results, NHANES and Quest Diagnostics

PFAS NASEM summation results	NHANES, 2017–2020, *n* = 3072	Quest Diagnostics, 2023–2024, *n* = 1023
NASEM summation median (average), highest observed, ng/mL	7.74 (10.65), 124.64	4.65 (5.96), 63.8
NASEM summed PFAS serum levels < 2 ng/mL	3.2%	15.3%
NASEM summed PFAS serum levels ≥ 2 ng/mL	96.8%	84.7%
NASEM summed PFAS serum levels 2– < 20 ng/mL	86.1%	82.2%
NASEM summed PFAS serum levels ≥ 20 ng/mL	10.7%	2.5%

## Discussion

### Methodology

We developed and validated an LC–MS/MS confirmatory method to detect and quantify nine PFAS. Comparing to the CDC NHANES method for national PFAS prevalence study where only one transition is used, our confirmatory method followed the standard for identification criteria in ANSI/ASB, including chromatographic separation, two precursor product ion transitions, and at least one ion ratio measured. For clinical laboratories, run time in this method was optimized to meet the requirement of high throughput and quick turnaround time. This method evaluated closely related structural isomers of PFOS and PFOA, in human serum and plasma specimens. To our knowledge, this is the first study that evaluates the prevalence and effect of all commercially available PFOS and PFOA isomer reference standards to help determine the prevalence of various isomers in authentic patient specimens.

Aligning with the NHANES method [13–15], PFOA branched isomer quantitation used br PFOA-P5. Robustness and accuracy were confirmed with br PFOA-P5 standalone certified reference materials. To better understand the capability of br PFOA-P5 in the calibrators to monitor other branched PFOA, standalone P3, P4, and P6 materials were tested; observations included varied response, accuracy bias, and failed ion ratio. We also purchased proficiency samples. Our results aligned with Centre de toxicologie du Québec samples. Ion ratio failures on patient samples with the quantifier calculated concentration above cutoff will be reported with an interference message and no quantitative value provided; these are interpreted as “null” results. Clinically, no positive results were observed when quantifying with either m/z 413/219 or 413/119 in authentic patient specimens for this isomer or other PFOA isomers in our study. For branched PFOS quantitation, our test offered enhancements on the NHANES method. Our method can accurately quantify the concentration of br PFOS-P4, P5, and P6 in patient specimens. Br PFOS-P5 and P6 quantitation requires both isomers to be used in calibrators with chromatographic separation (Fig. 1). The relative fragment ion responses (m/z 80 and 230) allow PFOS-P5 calibrators to be used to quantify PFOS-P4. In addition, the commonly observed interference caused by PFOS-P1 on linear PFOS quantitation was mitigated by appropriate fragment ion selection and Q0D voltage adjustment, ultimately resulting in rare interference observations in the final method.

Post-testing summation is used to report concentrations of br PFOS-P5 and P6 together as branched PFOS. Reporting all isomers is challenging in clinical laboratories as reference standard solutions are not available for all isomers (e.g., br PFOS-P2 and br PFOA-P2). Chromatographic separation efficiency between isomers can vary based on the chemical structures and characteristics, and product ion fragmentation efficiency and abundance can differ in authentic patient specimens.

To summarize, this method can accurately quantify the concentration of br PFOS-P4/P5, br PFOS-P6, and linear PFOS in patient specimens. These data support individual isomer-specific analysis providing more accurate and informative data compared with quantitative analysis of these chemicals combined in an unresolved single peak.

### Clinical assessment

The described prevalence study is also the first to utilize the NASEM guidance as best practice to evaluate a series of clinical specimens. By providing physicians with a NASEM-recommended PFAS summation, this test can help guide follow-up for PFAS-affected individuals. Most specimens had PFAS NASEM summations of 2 to < 20 ng/mL indicating that exposure reduction and age-appropriate standard-of-care were appropriate for these individuals. Follow-up is suggested for those individuals with PFAS summations of ≥ 20 ng/mL (2.5% in our study, 10.7% in 2017–2020 NHANES) who may be at increased risk of PFAS-related adverse health effects.

We also found the median PFAS sum was 4.65 ng/mL in this study. The NHANES study in 2017–2020 observed a median PFAS sum of 7.74 ng/mL (*n* = 3072). The lower median we found may reflect a decline in population PFAS levels over time [13–16, 46], or sample population exposure differences.

This method measures the level of the nine PFAS analytes (ten including the two branched PFOS isomers) and provides the overall PFAS level in serum and plasma at a given time. As human exposure often involves a combination of PFAS, these test results will not be able to pinpoint or identify the PFAS source. Since PFAS toxicity levels are still being established and the related adverse health effects are still under investigation, there is currently no clinical diagnosis or treatment recommendations specific to elevated PFAS levels [47]. However, with the initial test results as a baseline, individuals can monitor the PFAS levels over time to evaluate trends.

PFAS analytical methods are complex as nearly all PFAS are branched to some degree. Earlier production of PFAS by electrochemical fluorination (ECF) resulted in a mixture of 20 to 30% of branched PFOS and 15 to 20% of PFOA with linear forms. Although recent production of PFAS by telomerization results in isomerically pure products, different branched isomers may also be present in the final products from the impure starting materials [48, 49]. In 2013–2014, the NHANES study began measuring branched isomers of PFOS and PFOA and summing concentrations to report total PFOS and PFOA. As PFOS and PFOA are the most prevalent and abundant PFAS compounds, current clinical guidance from NASEM requires reporting branched PFOS and PFOA in addition to the linear isomers. Measuring both is informative because branched isomers may be eliminated from the body more rapidly than linear isomers [50–52] and, in some cases, may help identify source contributions [53–56]. In our study, the median (average) ratio percentage of branched PFOS to linear PFOS was 59.4% (64.7%); however, the range was 7.5 to 390%, demonstrating the necessity of monitoring both branched and linear PFOS in human serum as different patient populations may have more exposure to one form than the other.

With bioaccumulation of PFAS exposures in humans, this method is the starting point of monitoring PFAS exposure in clinical patient specimens for the currently recommended PFAS species. Due to exposure risk and the likelihood of detrimental impacts, PFOS and PFOA have both come under regulatory scrutiny, and many countries have restricted their use. However, PFAS challenges continue as the total number of PFAS species is still growing, new PFAS compounds are discovered, and many are under-recognized. More than 12,000 different PFAS compounds have been identified [56–61]. As global regulations restrict the use of legacy PFAS compounds, manufacturers are developing new PFAS, such as hexafluoropylene oxide dimer acid (HFPO-DA), also known as GenX. The environmental and health impacts of these new species are not understood. Most manufacturers have begun substituting perfluorooctyl-based products with perfluorinated chains of 4 and 6 carbons in length [62–65]. Compared to PFOS and PFOA, little is known about their replacements (such as PFBS and GenX). In April 2024, EPA announced *Final PFAS National Primary Drinking Water Regulation* which lists the maximum contaminant level (MCL) for PFOA and PFOS at 4 ppt for each, and for PFHxS, GenX, and PFNA, a 10 ppt each MCL [66]. Beyond the PFAS measure by EPA methods, comprehensive studies monitoring more PFAS from drinking water, dust samples, urine, serum, and whole blood specimens [67–70] provided some interesting correlation data between paired environmental samples and human specimens and revealed the specific distribution of some PFAS (e.g., FOSA found in urine and whole blood but not serum). For clinical laboratories, we are actively monitoring the new circulating PFAS in environments, working to expand PFAS testing in new matrices and expanding the PFAS panel with new recommendation and guidance released.

## Conclusion

We have developed and validated an LC–MS/MS method to detect and quantify nine PFAS, including closely related structural isomers, in human serum and plasma specimens in accordance with NASEM and CDC guidance. Furthermore, the prevalence was determined for the various PFAS and total PFAS levels in > 1000 clinical specimens. The median PFAS levels were lower than previously reported in large-scale studies, suggesting that PFAS levels may be declining on a population basis, likely attributable to the cessation of manufacturing of the most common PFAS. This quantitative serum PFAS method is useful to support addressing global PFAS challenges, monitor long-term PFAS exposure, provide data to correlate PFAS levels with health outcomes, and evaluate PFAS remediation in heavily exposed individuals and communities.

## Supplementary Information

Below is the link to the electronic supplementary material.

Supplementary file1 (DOCX 34 KB)
