# Supplementary material for: Development, validation, and clinical assessment of a liquid chromatography-tandem mass spectrometry serum assay for per- and polyfluoroalkyl substances (PFAS) recommended by the National Academies of Science, Engineering, and Medicine (NASEM)
Source: Anal Bioanal Chem. 2024 Sep 13;416(28):6333–44. doi: 10.1007/s00216-024-05519-y (PMC11541307; doi:10.1007/s00216-024-05519-y)
Supplement: Supplementary file 1 — Supplementary file1 (DOCX 34 KB) [file 216_2024_5519_MOESM1_ESM.docx]

**Table S1.** **PFAS compounds and labeled internal standard list with compound acronym, full name and product name**

| **Compound Acronym** | **Compound Full Name** | **Product Name (Wellington Laboratories)** |
| --- | --- | --- |
| PFOA | Perfluoro-n-octanoic acid | PFOA |
| PFNA | Perfluoro-n-nonanoic acid | PFNA |
| PFDA | Perfluoro-n-decanoic acid | PFDA |
| PFUndA | Perfluoro-n-undecanoic acid | PFUdA |
| PFHxS | Sodium perfluoro-1-hexanesulfonate | L-PFHxS |
| linear PFOS | Sodium perfluoro-1-octanesulfonate | L-PFOS |
| MeFOSAA | N-methylperfluoro-1-octanesulfonamidoacetic acid | N-MeFOSAA |
| branched PFOS-P6, br PFOS-P6 | Sodium perfluoro-6-methylheptanesulfonate | NaP6MHpS |
| branched PFOS-P5, br PFOS-P5 | Perfluoro-5-methylheptane sulfonate | P5MHpS (mixture with P5MHpA) |
| branched PFOA-P5, br PFOA-P5 | Perfluoro-5-methylheptanoic acid |  |
| MPFOA | Perfluoro-n-(1,2,3,4-^13^C4)octanoic acid | MPFOA |
| MPFNA | Perfluoro-n-(1,2,3,4,5-^13^C5)nonanoic acid | MPFNA |
| MPFDA | Perfluoro-n-(1,2-^13^C2)decanoic acid | MPFDA |
| MPFUnDA | Perfluoro-n-(1,2-^13^C2)undecanoic acid | MPFUdA |
| MPFHxS | Sodium perfluoro-1-hexane(^18^O2)sulfonate | MPFHxS |
| MPFOS | Sodium perfluoro-1-(1,2,3,4-^13^C4)octanesulfonate | MPFOS |
| d3-N-MeFOSAA | N-methyl-d3-perfluoro-1-octanesulfonamidoacetic acid | d3-N-MeFOSAA |

**Table S2: Non-related interference list challenged at 1,000 ng/mL in negative and PFAS LOQ serum samples**

| 1R,2S(-)-Ephedrine | Demoxepam | Meprobamate | Phenobarbital |
| --- | --- | --- | --- |
| 1S,2R(+)-Ephedrine | Desipramine | Metformin | Phentermine |
| 2-fluoroamphetamine | Desmethyldoxepin | Methadone | Phenylephrine |
| 2-fluoropmethamphetamine | Dextromethorphan | Methamphetamine | Phenylpropanolamine |
| 4-fluoroamphetamine | Dextrorphan | Methylphenidate | Phenytoin |
| 4-fluoromethamphetamine | Diazepam | Metoprolol | Pregabalin |
| 6-Acetylmorphine | Dihydrocodeine | Midazolam | Propoxyphene |
| 6B-Naltrexol | Diphenhydramine | Mitragynine | Propranolol |
| Acetaminophen | Doxepin | Morphine | Pyrilamine |
| Alphahydroxyalprazolam | Doxylamine | Naloxegol | Quinine |
| Alphahydroxytriazolam | EDDP | Naloxone | R,R(-)-Pseudoephedrine |
| Alprazolam | Fentanyl | Naltrexone | Ritalinic Acid |
| Aminoclonazepam | Fluconazole | Naproxen | Rufinamide |
| Amitriptyline | Flunitrazepam | Nicotine | S,S(+)-Pseudoephedrine |
| Amobarbital | Fluoxetine | Nitrazepam | Salicylic Acid |
| Amoxapine | Flurazepam | Norbuprenorphine | Secobarbital |
| Amphetamine | Gabapentin | Norcodeine | Sertraline |
| Atenolol | Hydrochlorothiazide | Nordiazepam | Tapentadol |
| Benzoylecgonine | Hydrocodone | Norfentanyl | Temazepam |
| Brompheniramine | Hydromorphone | Norhydrocodone | Theophylline |
| Buprenorphine | Hydroxybupropion | Norketamine | Topiramate |
| Butalbital | Hydroxyethylflurazepam | Normeperidine | Tramadol |
| Caffeine | Hydroxymidazolam | Normorphine | Trazodone |
| Cannabidiol | Ibuprofen | Noroxycodone | Triazolam |
| Cannabinol | Imipramine | Noroxymorphone | Trimipramine |
| Carisoprodol | Ketamine | Norsertraline | Valproic Acid |
| Chlordiazepoxide | Lacosamide | Nortriptyline | Vigabatrin |
| Chlorpheniramine | Lamotrigine | O-Desmethylvenlafaxine | Warfarin |
| Cimetidine | Levetiracetam | Omeprazole | Zolpidem |
| Citalopram | Levorphanol | Oxazepam | Zolpidem Metabolite |
| Clomipramine | Lidocaine | Oxcarbazepine | Zonisamide |
| Clonazepam | Lorazepam | Oxycodone | Zopiclone |
| Cocaethylene | l-Thyroxine | Oxymorphone | Zopiclone-N-Oxide |
| Cocaine | MDA | Pentazocine | Δ9-THC |
| Codeine | MDEA | Pentobarbital | Δ9-THCCOOH |
| Cotinine | MDMA | Phencyclidine |  |
| Cyclobenzaprine | Meperidine | Phenethylamine |  |

Note: list was sorted alphabetically.

**Table S3. PFAS-related interference list challenged at 10 ng/mL in negative and PFAS LOQ serum samples with compound acronym, full name and product name**

| **Compound Acronym** | **Compound Full Name** | **Product Name (Wellington Laboratories)** |
| --- | --- | --- |
| branched PFOS, br PFOS-P3 | Sodium perfluoro-3-methylheptanesulfonate | NaP3MHpS |
| branched PFOS, br PFOS-P1 | Perfluoro-1-methylheptane sulfonate | P1MHpS |
| branched PFOS, br PFOS-P3 | Perfluoro-3-methylheptane sulfonate | P3MHpS (mixture with P3MHpA) |
| branched PFOA, br PFOA-P3 | Perfluoro-3-methylheptanoic acid |  |
| branched PFOS, br PFOS-P4 | Perfluoro-4-methylheptane sulfonate | P4MHpS (mixture with P4MHpA) |
| branched PFOA, br PFOA-P4 | Perfluoro-4-methylheptanoic acid |  |
| branched PFOS, br PFOS-P6 | Perfluoro-6-methylheptane sulfonate | P6MHpS (mixture with P6MHpA) |
| branched PFOA, br PFOA-P6 | Perfluoro-6-methylheptanoic acid |  |
| branched PFOS, br PFOS-P55 | Perfluoro-5,5-dimethylhexane sulfonate | P55DMHxS (mixture with P55DMHxS) |
| branched PFOA, br PFOA-P55 | Perfluoro-5,5-dimethylhexanoic acid |  |
| branched PFOS, br PFOS-P45 | Perfluoro-4,5-dimethylhexane sulfonate | P45DMHxS (mixture with P45DMHxA, P35DMHxS, P35DMHxA) |
| branched PFOA, br PFOA-P45 | Perfluoro-4,5-dimethylhexanoic acid |  |
| branched PFOS, br PFOS-P35 | Perfluoro-3,5-dimethylhexane sulfonate |  |
| branched PFOA, br PFOA-P35 | Perfluoro-3,5-dimethylhexanoic acid |  |
| ipPFNA | Perfluoro-7-methyloctanoic acid | ipPFNA |
| ipPFNS | Sodium perfluoro-7-methyloctanesulfonate | ipPFNS |
| PFDoA | Perfluoro-n-dodecanoic acid | PFDoA |
| PFHpA | Perfluoro-n-heptanoic acid | PFHpA |
| PFHxA | Perfluoro-n-hexanoic acid | PFHxA |
| ADONA | Sodium dodecafluoro-3H-4,8-dioxanonanoate | NaDONA |
| FOSA-I | Perfluoro-1-octanesulfonamide | FOSA-I |
| L-PFBS | Potassium perfluoro-1-butanesulfonate | L-PFBS |
| L-PFHpS | Sodium perfluoro-1-heptanesulfonate | L-PFHpS |
| PFODA | Perfluoro-n-octadecanoic acid | PFODA |
| N-EtFOSAA | N-ethylperfluoro-1-octanesulfonamidoacetic acid | N-EtFOSAA |
| P4MOA | Perfluoro-4-methyloctanoic acid | P4MOA |
| P355TMHxA | Perfluoro-3,5,5-trimethylhexanoic acid | P355TMHxA |
| P37DMOA | Perfluoro-3,7-dimethyloctanoic acid | P37DMOA |
| T-PFOA | Technical Ammonium Perfluorooctanoate | T-PFOA |
| T-PFOS | Potassium perfluorooctanesulfonate | T-PFOS |
| br-PFHxSK | L-PFHxS with branched isomers (Potassium Salt) | br-PFHxSK |
| br-PFOA | Perfluorooctanoic acid with branched isomers | br-PFOA |
| br-PFOSK | L-PFOSK with branched isomers | br-PFOSK |
| br-PFNA | Perfluorononanoic acid with branched isomers | br-PFNA |
| br-NMeFOSAA | N-methylperfluoro-1-octanesulfonamidoacetic acid isomeric mixture | br-NMeFOSAA |
| br-NEtFOSAA | N-ethylperfluoro-1-octanesulfonamidoacetic acid isomeric mixture | br-NEtFOSAA |
| PFPeA | Perfluoro-n-pentanoic acid | PFPeA |
| PFTrDA | Perfluoro-n-tridecanoic acid | PFTrDA |
| PFTeDA | Perfluoro-n-tetradecanoic acid | PFTeDA |
| PFHxDA | Perfluoro-n-hexadecanoic acid | PFHxDA |
| L-PFNS | Sodium perfluoro-1-nonanesulfonate | L-PFNS |
| L-PFDS | Sodium perfluoro-1-decanesulfonate | L-PFDS |
| L-PFDoS | Sodium perfluoro-1-dodecanesulfonate | L-PFDoS |
| 4:2FTS | Sodium 1H,1H,2H,2H-perfluorohexanesulfonate *(4:2)* | 4:2FTS |
| 6:2FTS | Sodium 1H,1H,2H,2H-perfluorooctanesulfonate *(6:2)* | 6:2FTS |
| 8:2FTS | Sodium 1H,1H,2H,2H-perfluorodecanesulfonate *(8:2)* | 8:2FTS |
| GenX | 2,3,3,3-Tetrafluoro-2-(1,1,2,2,3,3,3-heptafluoropropoxy)propanoic acid | HFPO-DA |
| L-PFPrS | Sodium perfluoro-1-propanesulfonate | L-PFPrS |
| L-PFUdS | Sodium perfluoro-1-undecanesulfonate | L-PFUdS |
| L-PFTrDS | Sodium perfluoro-1-tridecanesulfonate | L-PFTrDS |
| PFAC-24PAR | Native PFAS Precision and Recovery Standard Solution | PFAC-24PAR |
| PFAC-MXJ | Native X:3 Fluorotelomer Carboxylic Acid Solution/Mixture | PFAC-MXJ |

**Table S4. Matrix effect study summary: difference (%) between the water diluted results and the non-diluted results as target**

| **PFAS** | **Low level** | | **Average for low level** | **High Level** | | | | | | **Average for high level** |
| --- | --- | --- | --- | --- | --- | --- | --- | --- | --- | --- |
|  | **Patient 1** | **Patient 2** |  | **Patient 3** | | | **Patient 4** | | |  |
|  | **2x** | **2x** |  | 2x | 5x | 10x | 2x | 5x | 10x |  |
| PFHxS | 2.30% | 0.37% | 1.33% | 1.58% | -1.99% | -1.23% | -1.99% | -5.09% | -5.29% | -2.34% |
| linear PFOS | -0.57% | 0.81% | 0.12% | -4.45% | -7.45% | -8.28% | -4.51% | -9.25% | -9.56% | -7.25% |
| br PFOS-P5 | -1.24% | -1.01% | -1.12% | -1.91% | -2.77% | -2.64% | -4.17% | -6.07% | -5.82% | -3.90% |
| br PFOS-P6 | 4.61% | 2.11% | 3.36% | -5.37% | -6.76% | -6.62% | -2.32% | -8.51% | -8.24% | -6.30% |
| linear PFOA | 5.06% | 8.35% | 6.71% | -7.28% | -1.63% | -9.72% | -5.42% | -13.28% | -8.42% | -7.62% |
| br PFOA-P5 | -3.34% | -3.53% | -3.43% | -8.22% | -4.05% | -10.84% | -0.72% | -3.13% | 0.46% | -4.42% |
| PFNA | -6.99% | -0.65% | -3.82% | -2.52% | -10.83% | -10.96% | -4.57% | -8.50% | -5.53% | -7.15% |
| MeFOSAA | -1.05% | -1.24% | -1.14% | -4.20% | -8.55% | -7.45% | -4.16% | -7.75% | -5.05% | -6.19% |
| PFDA | -3.20% | 0.48% | -1.36% | -8.57% | -8.68% | -13.51% | -7.13% | -8.61% | -7.75% | -9.04% |
| PFUnDA | -1.44% | 8.02% | 3.29% | -17.37% | -16.18% | -18.03% | -8.02% | -2.66% | -4.98% | -11.21% |

**Table S5. Serum and plasma specimen storage stability study : difference (%) of the results on day 90 as testing end point comparing to day 0 as the starting point with specimens stored at room temperature, refrigerated and frozen, 3 days post extraction stability and 3 freeze-thaw cycle stability**

| **PFAS** | **Room temperature**  **(15 to 30 °C)** | | **Refrigerated**  **(2 to 8 °C)** | | **Frozen**  **(-10 to -30 °C)** | | **3 days Post Extraction Stability**  **(15 to 30 °C)** | | **3 Freeze-Thaw Cycle Stability** | |
| --- | --- | --- | --- | --- | --- | --- | --- | --- | --- | --- |
|  | **Serum** | **Plasma** | **Serum** | **Plasma** | **Serum** | **Plasma** | **Serum** | **Plasma** | **Serum** | **Plasma** |
| PFHxS | 0.73% | -1.28% | -1.47% | -2.99% | -2.20% | -1.57% | -1.31% | 0.28% | 2.94% | -3.77% |
| linear PFOS | -2.46% | -1.72% | -4.50% | -1.32% | -4.37% | 0.26% | -1.91% | 0.52% | 1.36% | -2.60% |
| br PFOS-P5 | 0.60% | -4.00% | -2.40% | -4.57% | -2.10% | 0.29% | 1.80% | -0.28% | 2.10% | -0.28% |
| br PFOS-P6 | -3.13% | -6.75% | -4.12% | -7.30% | -4.45% | -4.87% | 2.64% | -1.08% | 1.15% | -4.92% |
| linear PFOA | 0.87% | 2.03% | -1.46% | 3.91% | -0.73% | 5.35% | -1.16% | 1.27% | 3.35% | -1.27% |
| br PFOA-P5 | -1.28% | -1.29% | -3.04% | 3.39% | -2.40% | 3.23% | -2.24% | 0.95% | 4.49% | 0.16% |
| PFNA | -0.78% | 0.47% | -0.78% | 1.74% | -2.82% | 3.01% | 0.16% | 1.55% | 1.57% | 1.24% |
| MeFOSAA | -2.86% | -1.84% | -2.86% | 0.67% | -3.87% | 1.50% | -0.67% | -1.14% | 2.35% | -2.12% |
| PFDA | 0.80% | -0.33% | -0.16% | 1.95% | -0.48% | 2.44% | -2.56% | -0.48% | -0.96% | -0.48% |
